# Readiness of Allied Professionals to Join the Mental Health Workforce: A Qualitative Evaluation of Trained Lay Trauma Counsellors’ Experiences When Refugee Youth Disclose Suicidal Ideation

**DOI:** 10.3390/ijerph18041486

**Published:** 2021-02-04

**Authors:** Sandra Löfving Gupta, Katarina Wijk, Georgina Warner, Anna Sarkadi

**Affiliations:** 1 Department of Public Health and Caring Sciences, Uppsala University, Box 564, 751 22 Uppsala, Sweden; Katarina.wijk@regiongavleborg.se (K.W.); georgina.warner@pubcare.uu.se (G.W.); anna.sarkadi@pubcare.uu.se (A.S.); 2 Centre for Research and Development, Region Gävleborg/Uppsala University, 801 87 Gävle, Sweden; 3 Department of Occupational Health Sciences and Psychology, Faculty of Health and Occupational Studies, University of Gävle, 801 76 Gävle, Sweden

**Keywords:** workforce solution, mental health workforce, trained lay counsellors, unaccompanied refugee minors, teaching recovery techniques, cognitive behaviour therapy, group intervention, stepped care model

## Abstract

The recent refugee crisis presented a huge challenge for the Swedish mental health workforce. Hence, innovative mental health workforce solutions were needed. Unaccompanied refugee minors (URM) are a particularly vulnerable refugee group. Teaching Recovery Techniques (TRT) was introduced as a community-based intervention utilising trained lay counsellors in a stepped model of care for refugee youth experiencing trauma symptoms. Professionals (e.g., teachers, social workers) can deliver the Cognitive Behavioural Therapy-based intervention after a brief training. A point of debate in this workforce solution is the readiness of trained lay counsellors to deal with potentially demanding situations like disclosure of suicidal ideation. This study aimed to explore the TRT trained lay counsellors’ experiences of procedures upon URM’s disclosure of suicidal ideation. Individual semi-structured interviews with TRT trained lay counsellors were conducted, then analysed using systemic text condensation. The analysis revealed four themes: “Importance of safety structures”, “Collaboration is key”, “Let sleeping dogs lie” and “Going the extra mile”. Dealing with suicidal ideation is challenging and feelings of helplessness occur. Adding adequate supervision and specific training on suicidal ideation using role play is recommended. Collaboration between agencies and key stakeholders is essential when targeting refugee mental health in a stepped care model.

## 1. Introduction

In 2016, Europe faced the largest single influx of refugees since World War II. This put a high demand on European countries to re-examine and find new sustainable solutions in various aspects of society, including the health care system. In Sweden, the country in the European Union with the highest number of asylum seekers per capita [1], the mental health service gap for this vulnerable group became evident. A substantial group of refugees were unaccompanied refugee minors (URM) [2], who still remain in Sweden. They have been described as the most vulnerable refugee group [3]. It has been formally acknowledged by the Swedish Social Services that existing psychiatric services do not meet the needs of this population, and an innovative mental health workforce solution is required to bridge the service gap [4].

### 1.1. Population Need

Numerous refugee children have been exposed to traumatic events like violence, threat, and separation in their country of origin and during migration [5]. URM, however, have a higher prevalence of traumatic events, such as torture, sexual abuse and kidnapping, compared to children fleeing with caregivers [6]. URM face the uncertainties of a complex asylum-seeking process and the stress of resettlement and acculturation hassles without the support of their caregivers [5,7]. Subsequently, trauma-related mental health problems, such as post-traumatic stress disorder (PTSD), depression, and anxiety, are particularly common among URM [8]. A recent study from Sweden [9] showed that 76% screened positive for PTSD, and a Norwegian study concluded that 43% met the criteria for a psychiatric diagnosis shortly after arriving at the host country [10]. Longitudinal studies confirm that these mental health problems are long-lasting [11,12]. PTSD diagnosis is associated with suicidality and this association is even stronger when there is comorbid depression. In Sweden, the rate of completed suicides among URM is almost 10 times higher compared to Swedish residents the same age [13]. A majority of URM live at residential homes, in absence of parents in the country, and are appointed a legal guardian. Immigration statistics indicate that the majority of URM who sought asylum in Sweden in 2015 were boys (86%) mainly from Afghanistan, Syria, Somalia and Eritrea between the ages of 13–17 [14].

### 1.2. Introducing a Stepped Care Model

A potential solution to bridge the mental health service gap is to bring allied professionals, such as teachers, nurses or social workers, into the mental health workforce, acting as a “first line of defence” in a stepped care model. Stepped care is a system of delivering and monitoring treatments. Patients start with an evidence-based treatment of low intensity as a first step and those who do not respond adequately “step up” to a subsequent treatment of higher intensity [15].

In 2016, Teaching Recovery Techniques (TRT) was introduced to Sweden. TRT is a Trauma-Focused Cognitive Behavioural Therapy (TF-CBT) manualised group intervention aimed towards children and adolescents with symptoms of PTSD [16]. It was designed to meet the needs of low-resource and community settings. TRT comprises two sessions for caregivers and five sessions for youth and includes the following components: psychoeducation, affective modulation skills, cognitive coping and processing, in vivo mastery of trauma reminders, guided exposure and exploring plans and hopes for the future. The first two sessions focus on intrusion, the third on arousal and the final two sessions deal with exposure. Among others, techniques like positive self-talk, dual attention and relaxation are used. The focus is on symptoms and tools rather than trauma narrative and processing. In addition, normalisation of trauma symptoms in the group environment is assumed to relieve youth from shame and fear, whereas the safe environment provided by caring adults is geared to rebuild youth’s trust in the adult world and provide social support. TRT in Sweden is delivered once a week during a seven-week period and two TRT facilitators co-host each session [17]. In Sweden, an introductory “getting to know each other session” prior to the core TRT sessions and a consolidating session at the end have been added. The purpose of the intervention and a brief overview of the content is discussed in the introductory session to help set expectations for the intervention. It is during this session that the TRT facilitators introduce themselves but there is no specific guidance upon how to do this. However, the importance of creating a safe environment in the group is stressed. When URM in Sweden were asked to describe their experience with the TRT intervention in a qualitative study the following six categories were revealed: social support, normalisation, valuable tools, comprehensibility, manageability and meaningfulness [9].

In order to become a TRT facilitator a three-day training workshop, run by the Swedish non-governmental organisation Children’s Rights in Society, is mandatory. Another requirement is that the facilitator meets refugee youth in their professional capacity. Facilitators may be psychologists or counsellors, but also staff with no previous therapeutic experience or specialist training in psychiatry are eligible to deliver the intervention after the workshop. Hence, some TRT facilitators could be referred to as “TRT trained lay counsellors” as they indeed have training in TRT but lack training in mental health or counselling albeit they might have professional training in other domains, such as teaching, nursing or social work.

**Figure 1:**
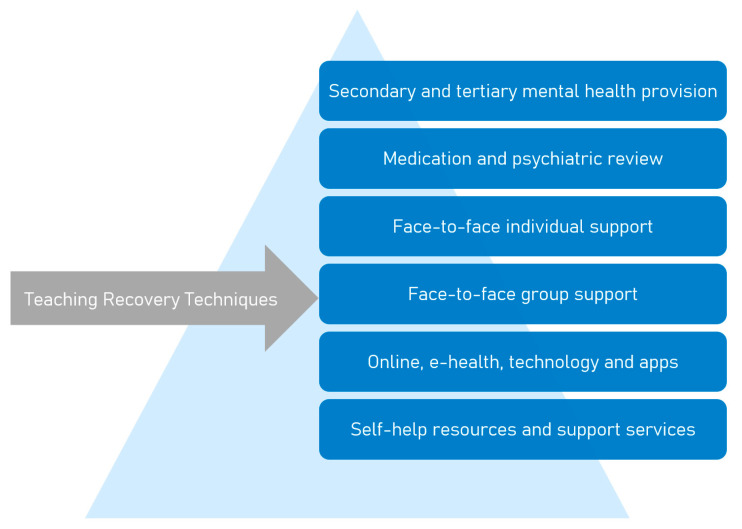
Placement of teaching recovery techniques in a stepped care model for mental health provision.

Given its brevity (seven weekly sessions), group format and delivery by community professionals, TRT offers several potential economies over individual therapy. Therefore, if a strong evidence base for TRT effectiveness can be presented, it is logical that the intervention would form a valuable component in a stepped care model (see Figure 1). International studies have reported high acceptability and large effect sizes for decrease in symptoms of both depression and PTSD [18,19]. An exploratory trial of TRT with 46 URM in Sweden (mainly male, ages 13–18) showed a significant decrease in reported symptoms of depression and PTSD [9]. There is an ongoing nationwide randomised control trial targeting URM in Sweden to further strengthen the evidence-base; as well as investigating the overall effectiveness, this trail aims to assess effectiveness at the subgroup level [17]. The URM are screened in schools, support groups and at residential homes using the Children’s Revised Impact of Events Scale (CRIES-8), where a score of 17 or above indicates high symptom burden and risk of PTSD [20,21] which makes them eligible for TRT.

Yet, a point of debate is the readiness and capability of allied professionals, such as teachers, nurses or social workers, to take on potentially demanding aspects of mental health intervention such as safety processes relating to suicidal ideation. Although a growing number of studies concerning CBT-based PTSD treatments delivered by lay counsellors [22,23,24] indicate feasibility and short-term effectiveness, there has been little exploration of trained lay counsellors’ experiences of safety procedures.

### 1.3. Safety Aspects and the Potential Vulnerability of TRT Trained Lay Counsellors

**Figure 2:**
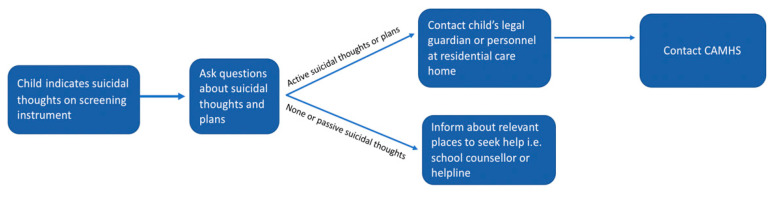
Teaching recovery techniques safety procedure.

Although TRT is not developed for a specialist health care setting, a substantial number of URM within the TRT program report severe, high-risk psychiatric symptoms. A pilot study (*N* = 55) showed that in addition to posttraumatic stress symptoms, 83% of URM receiving TRT suffered from moderate to severe depression and 48% displayed suicidal ideation or plans [9]. A safety protocol is recommended when dealing with people at risk. Safety protocols may vary depending on location, resources and infrastructure; however, it should clearly state how to assess risk, identify warning signs, implement safety planning techniques and when and how to refer the person at risk [25]. The questionnaires used in the TRT safety protocol have changed over time, with the Montgomery–Asberg Depression Scale MADRS [26] later replaced with the Patient Health Questionnaire PHQ-9 [27] and the Columbia Suicide Severity Rating Scale Screener, C-SSRS screener [28] introduced as a structured way to discuss suicidal thoughts, yet the overall process has remained the same (see Figure 2).

Although the TRT facilitators are not expected to do a full suicide risk assessment or to have knowledge about risk factors for suicide, as this responsibility lays within the Child and Adolescent Mental Health Service (CAMHS), they still need to ask potentially difficult questions about suicide and help decide whether the legal guardian should contact CAMHS or not. This may leave the trained lay counsellors, with no formal training in mental health or training on how to address suicidal thoughts, in a vulnerable and potentially risky situation. Although it is important to find new health care solutions to address a need in the community, in this case by task shifting from mental health professionals to allied professionals, it is essential that the personnel are adequately prepared for the task. Previous studies regarding lay counsellors delivering trauma-focused therapy have shown that training and practicing as a lay counsellor can enhance self-esteem and lead to empowerment, whereas others highlighted the particular stress, risk of overinvolvement and even risk of indirect traumatisation lay counsellors face [29,30,31,32]. However, these studies are conducted in low-income settings and in some cases the lay counsellors are recruited within the target community of the intervention and were not sufficiently trained. The importance of regular supervision has been raised by lay counsellors working with mental health interventions in previous qualitative studies [23,33].

Dealing with suicidal ideation and conducting suicide risk assessments is a complex and challenging task that even trained mental health personnel struggle with and feel anxious about [34,35]. It is of great importance to learn about the experiences and potential struggles of lay counsellors when they are exposed to someone with suicidal thoughts or plans. This knowledge is crucial to give adequate support and training to the lay counsellors and, ultimately, to ensure the persons signalling suicidal communication are given adequate care.

Hence, the aim of this study was to examine how TRT facilitators, without formal training in mental health or counselling i.e., TRT trained lay counsellors, experience the safety procedure when participating unaccompanied refugee youth disclose suicidal ideation.

## 2. Methods

### 2.1. Data Collection

National recruitment of TRT facilitators was conducted through email within the Children’s Rights in Society network of operating TRT facilitators (*N* = 50). The study invitation email explained the purpose of the study and the inclusion criteria: TRT facilitators without therapeutic training or formal education in psychiatry (TRT trained lay counsellors) and who had experienced URM disclosing suicidal ideation during the TRT group session.

Recruitment continued until saturation was reached and no new information was observed in the data. Ten interviews, using an interview guide (Table 1), were conducted using different forms of communication; face to face, via Skype and by phone and varied in length between 35–45 min. Of these ten interviews, two were excluded from analysis. The first was a pilot interview and the other was excluded since the TRT facilitator had formal training in psychotherapy, contrary to inclusion criteria of no formal mental health training. All interviews were conducted by the first author who is a trained TRT facilitator and a child- and adolescent psychiatrist.

The included respondents (*N* = 8; 7 females and 1 male) were from all over Sweden, both rural and urban areas. They included two nurses, five social workers and a child welfare-officer. They had facilitated between 1–10 TRT groups each, with an average of three groups.

### 2.2. Ethical Considerations

After a pilot interview, it became evident that, although the respondents were interviewed in their professional capacity, which would not normally need ethical clearance according to Swedish legislation, the respondents’ disclosure was of such sensitive character that we reconsidered and obtained ethical approval (dnr 2019-01427), excluding the pilot interview from further analyses.

### 2.3. Data Analysis

**Figure 3:**
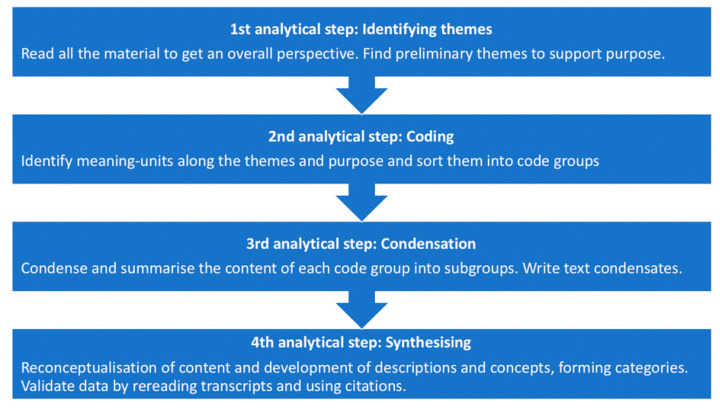
Analytical process of systematic text condensation (STC).

All interviews were transcribed verbatim and analysed using Systematic Text Condensation (STC) as described by Malterud [36] (see Figure 3 for a description of the analytic process of STC). STC was chosen for analysis for its descriptive and inductive approach that presents the experiences as described by the participants, rather than searching for underlying meaning. First, all the transcripts were read and re-read by all authors to obtain a full comprehension of each case. This was followed by the process of decontextualisation, in which, recurrent themes regarding the respondents’ different experience of dealing with URM who disclosed suicidal thoughts were recognized. Meaning units were then identified and grouped, these were subsequently sorted into categories describing different aspects of the themes. The content of each category was summarized and expressed as a single statement. The analytical text was formulated and quotes illustrating the different categories were selected. Finally, the results were validated by rereading all eight original transcripts to see whether the themes and code groups had goodness-of-fit.

## 3. Results

Four themes emerged during the analysis. The themes and their corresponding categories are presented in Table 2.

### 3.1. Theme 1: Importance of Safety Structures

The TRT lay counsellors recognized the significance of having pre-existing routines at the workplace but they also emphasized the importance of finding comfort in a colleague, someone at the workplace in whom they could confide when meeting URM who disclosed suicidal thoughts. Moreover, having established a safety protocol, a step by step guide on what actions to take when a URM discloses suicidal thoughts, was also considered helpful.

#### 3.1.1. Established Safety Routines at the Workplace

TRT lay counsellors working in a medical setting valued the well-defined safety structures and routines that were already established at the workplace. They stated that having a doctor available at the workplace, who was able to do a suicide assessment and refer to mental health services when needed, was very valuable. Although the doctor might not be available the same day as the URM made the disclosure, the mere knowledge that an assessment would be made by someone else was described as a relief. In some workplaces an experienced counsellor was available to assess and have regular individual follow ups with the URM in need. However, the social workers often did not have established safety structures in place as they did not have access to anyone with formal mental health training at their workplace nor did they have established pathways for referral when suicidal ideation was disclosed.

“I don’t feel that we overlook anything. Since we are at a healthcare centre. I feel that we already have a safety informed way of thinking…we are prepared to deal with this kind of (pause) [suicidal communication]. We haven’t felt scared that something will happen.”

(Interview 3)

#### 3.1.2. Comfort in Colleagues

The TRT lay counsellors also emphasised the importance of having a close and trustful relationship with a colleague, either the co-TRT facilitator or someone else at the workplace. When reflecting on features of this colleague the TRT lay counsellor did not attribute formal training in suicide assessments as a prominent feature. Rather, that the colleague was someone the TRT lay counsellor could confide in or someone who expresses confidence in situations dealing with suicidal disclosure.

“I have learnt so much by facilitating these groups. I don’t think I would have wanted to run a group with someone I did not feel secure with. The most important thing is that we know each other and that we can get through this together.”

(Interview 3)

The opposite was also highlighted, that not knowing the co-facilitator beforehand and the lack of trust and co-operation between the TRT facilitators was considered particularly stressful when meeting URM who had disclosed suicidal thoughts.

#### 3.1.3. Working with the Safety Protocol

Having a safety protocol was viewed as beneficial when dealing with URM who had disclosed suicidal ideation. TRT lay counsellors explained the safety protocol gave them structure and confidence. However, they also stated the safety protocol did not take into account the complexities involved in asking questions about suicide. They recall that even though they asked the suggested questions regarding suicidal thoughts/plans the answers were not clear, one URM even refused to answer the questions.

“It felt really good to have a safety protocol, it felt good to have something to lean against. It felt like a backbone. It was something we facilitators talked about beforehand. But in reality, it wasn’t as straightforward. There were many factors we couldn’t control…”

(Interview 4)

The more experienced TRT lay counsellors, who had seen different versions of the safety protocol, implied the earlier versions were too sensitive to suicidal ideation. In fact, one TRT lay counsellor reported that she consciously did not follow the initial protocol as she knew the URM and was not worried about him harming himself although he scored high on the screening.

### 3.2. Theme 2: Collaboration Is Key

Collaborating with CAMHS and sharing the same understanding about TRT as key persons in the URM’s network such as legal guardians and personnel at residential care homes was considered essential when dealing with URM who had disclosed suicidal ideation.

#### 3.2.1. Closed Doors to CAMHS

The TRT lay counsellors’ expectations and experiences of collaborating with CAMHS varied. Some reported their previous negative experiences led them to be concerned about this collaboration even before the TRT group started.

“That was something I thought about even during the training, you know, if we find someone with suicidal thoughts, will CAMHS agree to see them? Or will it just be nothing, limbo? And will I have time to handle it, if it ends in limbo?”

(Interview 1)

A number of TRT lay counsellors reported actual experiences of not receiving the help and care they had needed from CAMHS. There was a sense that CAMHS normalised the URMs’ symptoms and suicidal thoughts or did not offer an assessment as urgently as the TRT lay counsellor expected. This left the TRT lay counsellor with a sense of being alone and vulnerable.

“I felt that we did not get an adequate response [from CAMHS]. They did not take it seriously, like ‘this kid has gone through so many horrible things that he is expected to have suicidal thoughts’. They just normalised it and that was not good…it just ended there… and we were left to take care of it somehow.”

(Interview 4)

Although some TRT lay counsellor did not report any specific encounter with CAMHS it was clear they had low expectations of what specialist mental health services could offer. One TRT lay counsellor even recommended the legal guardian to seek help within primary healthcare instead of CAMHS due to prior problems within CAMHS.

“There have been huge problems with CAMHS in my city… it was chaos actually. Enormous waiting lists and on top of that it’s my experience that they don’t deal with things. So, yes, I have very low expectations.”

(Interview 2)

On the other hand, there were also TRT lay counsellors who shared a good experience of collaborating with CAMHS and felt adequately helped by CAMHS.

“I actually felt like they did a good job, it might take some time…But if someone is really unwell it might go faster… We had two or three who were in a really bad state and CAMHS agreed to see them.”

(Interview 5)

#### 3.2.2. Realising a Shared Understanding among Key Persons

The experiences of collaboration with legal guardians and personnel at residential care homes varied. Some facilitators experienced difficulties in this collaboration, for instance, practical difficulties to get in contact with the legal guardian when the URM had disclosed suicidal intention, which left the TRT lay counsellor with a huge responsibility.

“Some legal guardians were really good but it did not always work out well. They were difficult to get hold of. Maybe they did not fully understand their role? But some were really engaged. One legal guardian asked if some other teenagers that she knew could join the group. I think they appreciated that we actually did something.”

(Interview 6)

Some legal guardians and personnel for residential homes were openly opposed to and critical of TRT, since they felt TRT could potentially be harmful. One TRT lay counsellor mentioned that differences in educational level between the TRT lay counsellors and the personnel for resident care home led to misunderstanding, which was considered an obstacle.

“Some of the legal guardians had a negative reaction to what we did. Like, okay, now you have had this thing and you are just dumping it all on us. What are we supposed to do with this? We met the same reaction from the ones working at the residential care home. They felt that we stirred up too much among the teenagers and maybe we did? That we caused more harm than good.”

(Interview 8)

On the other hand, there are also examples of excellent collaboration where the key persons have been fully onboard, supportive and played a crucial role in for instance helping URM to practice TRT skills outside the sessions.

### 3.3. Theme 3: Let Sleeping Dogs Lie

Despite the safety protocol, some TRT lay counsellors felt insecure and personally responsible for discovering suicidal thoughts. Several TRT lay counsellors did not feel equipped to meet URM with mental ill health and suicidal ideation and there were TRT lay counsellors who wondered whether the intervention actually might have a negative effect on the URMs’ wellbeing.

#### 3.3.1. Navigating the Boundaries of Responsibility

The safety protocol clearly stated the TRT lay counsellors role when participating URM disclosed suicidal ideation. Yet, some TRT lay counsellors described that they felt personally responsible for being the one who discovered the suicidal thoughts and it was unsatisfactory for them to refer the URM to someone else. They expressed a need to go beyond the safety protocol.

“Of course, I sometimes felt like I should have been the one to be there for them. You know, instead of just leaving it to someone else. Do you see what I mean? That I wanted to see it through all the way. And we couldn’t do that… We left these people when we should have been the ones who made sure everything turned out okay.”

(Interview 5)

#### 3.3.2. Readiness to Talk About Suicide

Some TRT lay counsellors worried that they might lack the competence to assess the URM mental health and that they might disregard something important that the URM was signalling non-verbally. There were times when they were worried a participant actually might harm themselves or even commit suicide and they reflected on their personal responsibility, if something like that would happen.

“I can’t really pinpoint what I felt I needed, maybe more experience…I don’t know if I needed more experience of working with suicidal youth or just more life experience.”

(Interview 4)

“But it became easier with time, with more experience…you learn from your mistakes. But of course, sometimes I was really worried, what if they were to jump in front of a train? I would have needed someone to talk to there and then, and that was difficult.”

(Interview 6)

Others did not feel any discomfort or threat by the idea that asking questions about suicide might evoke strong negative emotions. Although they had not had any formal training in suicide assessments they felt prepared and safe to ask questions about suicide.

“I believe that many adults are afraid of asking questions about suicide because they think that it is better to let sleeping dogs lie. I thought that it was liberating to realize that it wasn’t the case. On the contrary, when you dare to address it the teenager actually reveals their thoughts. That is something every grown up who encounters teenagers needs to hear.”

(Interview 7)

#### 3.3.3. Notion of Doing Harm

Some TRT lay counsellors expressed worry and apprehension about the negative emotions that might be triggered by talking about traumatic memories. Sometimes they were challenged by a particular URM’s resistance to talk about trauma. One TRT lay counsellor reflected over that, even though she knew the importance of the exposure session, she felt inhibited to fully implement since she was not sure she could manage the emotions the exposure might trigger in the URM. However, the feeling of doing potential harm seemed to be greatest when conducting their first TRT group, as TRT lay counsellor describe this feeling decreased with time.

“I remember thinking many times, this is too much for me. What have I gotten myself into?… So, I thought, oh my God what kind of processes are we starting? Are we saying things no one else has said? We are talking about stuff and reviving their memories. What am I supposed to do with that? I don’t know exactly what I felt, maybe powerless?”

(Interview 4)

Others did not feel any discomfort or threat by the fact that the TRT session might evoke strong negative emotions. On the contrary, they normalised it and viewed it as a part of the process.

“Personally, I have never been afraid of meeting people with mental ill health on the contrary I find it quite interesting to see whom they choose to tell their story to.”

(Interview 5)

“I really believe that it is crucial to label things for what they are. I do not hesitate to say the difficult words.”

(Interview 1)

### 3.4. Theme 4: Going the Extra Mile

TRT lay counsellors described the need for a manual-based intervention for URM whom they had identified as a particularly vulnerable group in society. They also related to their own desire (and struggle) to find meaning and to be creative and flexible in an unstable and chaotic situation in order to deliver TRT.

#### 3.4.1. Motivated by a Structured Way of Addressing a Need

The TRT lay counsellors had identified URM to be a vulnerable group in need of coping strategies and knowledge about trauma and post-traumatic stress. They expressed frustration that URM did not access proper treatment elsewhere and craved an intervention that was hands-on and manual based. This was emphasized as an important motivational factor for joining the TRT training.

“I really felt that I needed to do something for this group because, you know, people said that there is nothing we can do as long as they are asylum seekers…it is better to do something than nothing.”

(Interview 6)

At the same time, a few TRT lay counsellor explained that facilitating TRT groups simply was a part of their job description and they had been asked to do the training by their supervisors and managers, rather than by their own identified need or conviction.

#### 3.4.2. Fitting into the Chaos

Meeting URM in a situation of crisis, where a number of basic needs such as shelter and food are not being met, placed high demands on the TRT lay counsellor and was sometimes challenging for them. Yet, the TRT lay counsellor have shown signs of both creativity and flexibility as they described various ways and strategies in which they sought to increase motivation, session attendance and to facilitate the URMs’ ability to fully engage in the sessions. This could entail seemingly small gestures like offering food during the sessions or to make wake-up phone calls to URM with sleeping difficulties. However, TRT lay counsellors reflected this “extra care”, outside the manual, felt important not only for the URMs’ wellbeing, but also for the lay counsellors themselves as it gave them a sense of accomplishment and meaning in a situation of chaos.

“Sometimes you need to go beyond the manual. You need to make sure that they come despite the fact that they are homeless. You need to offer fellowship, food and laughter. You need to try. It was difficult but you need to find meaning because, you know, we could offer them something.”

(Interview 1)

TRT lay counsellors also expressed great empathy and distress over the URMs’ vulnerable and often uncertain life situation relating to the stress of being in the asylum-seeking process. A few TRT lay counsellors questioned whether TRT and addressing trauma actually was suitable in this situation of uncertainty.

“They were mostly guys from Afghanistan…And they didn’t know if they were going to be granted asylum in Sweden. So, they were in the middle of the asylum-seeking process. This was a major thing for them, if they were going to be able to stay or not. So, this was the dominant thing for them—not thinking about trauma… I feel that the timing of this intervention was wrong.”

(Interview 8)

## 4. Discussion

This study explored how TRT trained lay counsellors, without formal training in mental health or counselling, experienced dealing with URM disclosing suicidal ideation.

Although some TRT lay counsellors felt anxious and overwhelmed by the disclosure of suicidal ideation, others were surprisingly confident. Dealing with suicidal disclosures seems to be a challenging task regardless of professional training. A recent qualitative study among psychiatrists in Sweden describes feelings of fear, anxiety, uncertainty and even physical reactions in relation to suicide risk assessment [34]. Although the TRT lay counsellors’ role regarding suicidal disclosure is not comparable with the role of a psychiatrist conducting a full suicide risk assessment, the experience of uncertainty, fear of making the wrong decision and sense of responsibility unites them. However, an interesting difference is while the TRT lay counsellors reflect over the boundaries of their moral responsibility, the psychiatrists also reveal being burdened by formal responsibility and the fear of malpractice litigation when assessing suicide [34] from which the TRT lay counsellor are spared. One may speculate that formally trained personnel with a legislative duty to, for instance, keep health records, might experience suicidal disclosure with regard to their professional responsibility differently than the lay counsellors.

While the TRT lay counsellors acknowledged the importance of safety structures and there was a general appreciation of the safety protocol and procedures, asking questions about suicide was not always straightforward. The TRT lay counsellors experienced that, despite having followed the instructions in the safety protocol, the URM refused to answer questions about suicide or that the URM was emotionally blunted which made therapeutic alliance difficult. There are several reasons for not disclosing suicidal thoughts, such as lack of trust, fear of hospitalisation, judgment or causing distress for the person asking the questions or even lack of empathy in the person asking the questions [37,38]. Context and proper training in giving rationale for asking questions about suicidal thoughts are important.

Furthermore, establishing interpersonal trust and setting aside screening questionnaires to strengthen therapeutic alliance has been positively correlated to greater overall disclosure [37]. Struggles to obtain suicidal disclosure is not unique to utilisation of lay counsellors or the community setting, it is also found in a therapeutic setting with formally trained and experienced therapists [39]. Similar challenges of issues related to lack of emotional contact and credibility has been reported by trained psychiatrists and adding additional training on understanding non-verbal signs that may signal increased suicide risk has been suggested [34].

The readiness to talk about suicide varied among the TRT lay counsellors. Some reported feelings of insecurity when dealing with suicidal disclosure and suggested lack of experience (referring to both work and life experience) as a possible explanation. This is in line with a Swedish study among trained personnel working within mental health, concluding that job clarity and confidence regarding their role with suicidal patients as well as attitude towards suicidal prevention was connected to work experience as well as perception of having received sufficient suicidal prevention education [40]. Adding roleplay to training e.g., has shown both reported and observed improvement in communication with youth in distress and directly asking questions about suicide [41] in the context of a community-based suicide prevention intervention. Enhancing the present TRT training with rationale for asking questions about suicidality, training on non-verbal signs and roleplay on asking questions about suicidality could be beneficial and evaluated in future research.

The URMs’ situation, their trauma narrative and helplessness in the asylum-seeking process were demanding for some TRT lay counsellors who felt great empathy but also overwhelmed and sometimes helpless themselves. However, they were also motivated by this challenging situation and experienced meaning in helping the specific URM reduce trauma symptoms and by being a part of influencing social injustice as well as addressing need. Even among experienced trauma counsellors, “providing assistance to others” both at a personal and societal level has been described as rewarding and as important factors to thrive as a trauma therapist [42]. Experienced trauma therapists also reflect over the need to modulate their own empathy and by setting boundaries and accepting the counselling intervention and their own limitation [42]. Supervision is important to support lay counsellors [23,43]; however, adequate supervision should include both management of clients and specifically inquiring about counsellors’ own emotions to address the particular risk of indirect traumatization [44]. A future research direction could be to design and evaluate a dedicated supervision program for TRT facilitators.

The TRT trained lay counsellors also revealed concerns regarding the tolerability and safety of exposure and the strong negative emotions exposure evoked. This fear of doing potential harm is not limited to lay counsellors. A study among 600 mental health workers disclosed similar concerns regarding exposure although some of them used exposure in their practice [45]. Despite the strong evidence of the efficacy of exposure, even trauma experts are afraid of exposure causing symptom exacerbation and drop outs, leading exposure techniques to be under used [46,47]. Though the TRT lay counsellors received brief training in the rationale for exposure and how to conduct exposure, adding enhanced emotion-based training targeting attitude change by identifying concerns about exposure and adding video-based client testimonies may reduce concerns and enhance delivery of exposure [48]. Given the strength of opinion coming through regarding exposure, more thorough investigation of this particular topic could be warranted.

The need for a shared understanding with key stakeholders was identified by the TRT lay counsellors, as some legal guardians and personnel from residential care homes questioned the need and purpose of the intervention as they feared the URM might experience more trauma symptoms due to the intervention. However, the TRT lay counsellors who reported most apprehension and resistance from other stakeholders also reported that they themselves became TRT facilitators due to the will of their managers rather than their own conviction of a need for an intervention. One might speculate that this group of lay counsellors were more sceptic to the intervention and therefore not as well equipped to explain the benefits or reduce misbeliefs regarding the intervention to the stakeholders. Despite efforts made to reduce the knowledge gap and stigma regarding psychiatric treatment in Sweden, a study on change in public attitudes regarding mental health concluded that appreciation of treatment of mental illness and psychiatric care remains low [49]. Hence, facing negative beliefs about mental health care is common and not unique to lay counsellors.

The TRT lay counsellors were also concerned about URM not being assessed or admitted to CAMHS. This perceived “closed door” to CAMHS could in part be due to a debated belief that trauma treatment should not commence in an unstable setting, traditionally excluding asylum seekers from accessing trauma treatment in specialist care [50]. Although CAMHS are obliged to conduct suicide risk assessments on asylum seekers, the way the profile of the population interacts with the existing service model of care is a potential source of friction in collaboration. This lack of well-functioning collaboration and accessibility to CAMHS left the lay counsellors with a sense of loneliness and vulnerability. This structural problem is in-line with a previous study emphasising “building relationships between agencies” and increasing accessibility to mental health services for refugee children are crucial for increased service utility [51] and needs to be addressed at a health governance level. There needs to be more time dedicated to identifying ways in which collaboration could be enhanced. One could look to the interdisciplinary collaboration literature [52] to look for attributes to target for instance, effective communication channels between CAMHS and TRT facilitators, shared accountability and building trust. The effectiveness of these working models in improving collaboration would need to be evaluated.

### Methodological Considerations

The first author is a child and adolescent psychiatrist with long experience of meeting URM and conducting suicide assessments at CAMHS. She is also a trained TRT facilitator and had, at the time of the interviews, conducted one TRT group. Although this background gave her great knowledge and experience in the field of psychiatry and suicide assessments, throughout the study there, has been an awareness of how this might impact the interviewed TRT lay counsellors, i.e., researcher reflexivity. There was an initial concern the TRT lay counsellors might feel intimidated or judged, less likely to reveal own limitations or less likely to speak freely about negative experiences of collaborating with CAMHS. In order to reduce her potential role as an ”expert”, or as a spokesperson for CAMHS, this issue was addressed before the interviews and by making extra efforts to create an interview environment that promoted trust and openness. In addition, the purpose of the study was stressed repeatedly and participants encouraged to speak freely and honestly.

To further strengthen credibility, a semi-structured interview guide was used. To promote transferability, interview data were collected from TRT lay counsellors with different occupations, working in different geographical areas in Sweden and a difference in number of conducted TRT groups. Furthermore, transferability was also promoted by describing both typical and atypical views expressed by the TRT lay counsellors within each theme, i.e., negative case analysis. Although the number of interviews could seem low, saturation was assessed, and all authors were in agreement that it had been reached. Dependability and confirmability were enhanced by having a clear research trail during the entire process of analysis and involving all authors in the analysis. Reflecting on possible preconceptions was an essential part in this process.

Limitations: This study adopted a qualitative methodology that intended to investigate experiences in the particular context of Sweden, hence generalizability of the findings to other international contexts was not an expected attribute; the structure of health and social care in the local context would need to be considered. The study also specifically addressed the target group of URM and not refugee adolescents in general. It is possible that knowing there is a parent available to contact and discuss with might alter the experiences of the lay counsellors. Finally, questions about suicidal ideation followed initial screening and were part of a safety protocol. Thus, the study does not cover situations where disclosure is spontaneous, not backed up with a plan. It is likely that those situations cause more apprehension, anxiety, and insecurity in lay counsellors. Future studies could therefore expand the scope of target groups to refugee or otherwise vulnerable adolescents and counselling types, to investigate how lay counsellors deal with disclosures of suicidal ideation.

## 5. Conclusions

Dealing with suicidal disclosure is a complex and challenging task regardless of training. Both lay counsellors and experienced mental health workers struggle with feelings of uncertainty, helplessness and boundaries of responsibility; however, lay counsellors seem to be exempt from fear of professional repercussions. The motivations for becoming a TRT facilitator might also be interacting with their perceived experiences. Adding specific training on how to address suicidal throughs and talk about suicidal ideation using roleplay is recommended. Adding “attitude change” based training specifically challenging the concerns regarding exposure could be valuable, as well as adequate supervision advising on management but also targeting the lay counsellors’ own emotions is recommended. Finally, collaboration with key stakeholders and building relationships between agencies is essential to facilitate working with refugee mental health in a stepped care model and new working models based on interdisciplinary collaboration is recommended. Another potential obstacle for collaboration regarding this particular population could be conflicting views about the timing of trauma treatment, which needs to be addressed. Overall, although there is room for improvement in training and collaborative working, incorporation of allied professionals in the mental health workforce appears to be a workable solution to the mental health needs of URM in Sweden.

## Figures and Tables

**Table 1:** Interview guide.

S/N	Questions
1	What motivated you to become a TRT facilitator?
2	Tell me what you remember about your feelings and thoughts during the TRT training. a. What were you enthusiastic about? b. Was there something that intimidated or worried you? c. Anything else?
3	Describe your previous experience of working with depressed or suicidal youth? a. Do you have any experience of suicidal youth outside your work, NGO? b. What about working with refugees?
4	Describe your experience of being a facilitator in TRT working with suicidal youth? a. How did you discover that they had suicidal thoughts? b. What did you do? c. What was helpful for you in your assessment?
5	How did you perceive the safety protocol?
6	How would you describe your experience of collaborating with the legal guardians concerning suicidal youth?
7	How would you describe the collaboration with mental health specialist services regarding suicidal youth? Please exemplify.
8	Do you feel that you had enough knowledge or experience to handle suicidal youth?
9	In your opinion, is there anything that can change within TRT training or program in order to facilitate for group leaders working with suicidal youth?

**Table 2:** Overview of themes and categories.

Themes	Categories
Importance of safety structures	Established safety structures at the workplace Comfort in colleagues Working with the safety protocol
Collaboration is key	Closed doors to child and adolescent mental health services Realising a shared understanding among key persons
Let sleeping dogs lie	Navigating the boundaries of responsibility Readiness to talk about suicide Notion of doing harm
Going the extra mile	Motivated by a structured way of addressing a need Fitting into the chaos

## Data Availability

The datasets generated and/or analysed during the current study are available from the corresponding author on reasonable request.
